# 3D interactive tractography-informed resting-state fMRI connectivity

**DOI:** 10.3389/fnins.2015.00275

**Published:** 2015-08-11

**Authors:** Maxime Chamberland, Michaël Bernier, David Fortin, Kevin Whittingstall, Maxime Descoteaux

**Affiliations:** ^1^Centre de Recherche CHUS, University of SherbrookeSherbrooke, QC, Canada; ^2^Sherbrooke Connectivity Imaging Lab, Computer Science Department, Faculty of Science, University of SherbrookeSherbrooke, QC, Canada; ^3^Department of Nuclear Medicine and Radiobiology, Faculty of Medicine and Health Science, University of SherbrookeSherbrooke, QC, Canada; ^4^Division of Neurosurgery and Neuro-Oncology, Faculty of Medicine and Health Science, University of SherbrookeSherbrooke, QC, Canada; ^5^Department of Diagnostic Radiology, Faculty of Medicine and Health Science, University of SherbrookeSherbrooke, QC, Canada

**Keywords:** diffusion MRI, resting-state fMRI, tractography, structure-function, variability, visualization

## Abstract

In the past decade, the fusion between diffusion magnetic resonance imaging (dMRI) and functional magnetic resonance imaging (fMRI) has opened the way for exploring structure-function relationships *in vivo*. As it stands, the common approach usually consists of analysing fMRI and dMRI datasets separately or using one to inform the other, such as using fMRI activation sites to reconstruct dMRI streamlines that interconnect them. Moreover, given the large inter-individual variability of the healthy human brain, it is possible that valuable information is lost when a fixed set of dMRI/fMRI analysis parameters such as threshold values are assumed constant across subjects. By allowing one to modify such parameters while viewing the results in real-time, one can begin to fully explore the sensitivity of structure-function relations and how they differ across brain areas and individuals. This is especially important when interpreting how structure-function relationships are altered in patients with neurological disorders, such as the presence of a tumor. In this study, we present and validate a novel approach to achieve this: First, we present an interactive method to generate and visualize tractography-driven resting-state functional connectivity, which reduces the bias introduced by seed size, shape and position. Next, we demonstrate that structural and functional reconstruction parameters explain a significant portion of intra- and inter-subject variability. Finally, we demonstrate how our proposed approach can be used in a neurosurgical planning context. We believe this approach will promote the exploration of structure-function relationships in a subject-specific aspect and will open new opportunities for connectomics.

## 1. Introduction

In the era of multi-modal magnetic resonance imaging (MRI), combining diffusion MRI (dMRI), and functional MRI (fMRI) permits a unique way of exploring structure-function relationships *in vivo*. With dMRI (Le Bihan and Breton, 1985; Le Bihan et al., 2001; Basser and Jones, 2002), it is possible to probe the microstructure of biological tissues such as white matter connections of the brain (i.e., structural connectivity). On the other hand, fMRI provides 4D whole-brain images that reflect changes in cortical blood flow, volume and oxygen as measured by the Blood-Oxygenation-Level-Dependant (BOLD) signal (Turner, 1992; Kwong et al., 1992; Bandettini et al., 1993). At rest, the spontaneous low frequency fluctuations (< 0.08–0.1 Hz) in the BOLD signal allow the detection of temporally correlated spatial patterns, also known as Resting State Networks (RSNs) (Biswal et al., 1995; Damoiseaux et al., 2006). The common way of exploring such RSNs is to extract the preprocessed BOLD time course from an a priori region of interest (ROI) and compute the temporal correlation with all other voxels of the brain. The result is a *seed-specific* correlation map or a functional connectivity map.

Integrating dMRI and fMRI is often necessary to understand how patterns of functional connectivity are related to structural connectivity. For instance, is functional connectivity present in the absence of structural connectivity, or is one predictive of the other (Honey et al., 2009)? While many studies have investigated this in the past (Honey et al., 2009; van den Heuvel et al., 2009; Mennes et al., 2010; Zhang et al., 2010; Várkuti et al., 2011; Bowman et al., 2012; Hermundstad et al., 2013; Sporns, 2013; Whittingstall et al., 2013; Goni et al., 2014; Ward et al., 2014; Zhu et al., 2014), surprisingly few studies have investigated how subtle changes in the analysis pipeline may alter structure-function relationships (Bastiani et al., 2012). For example, functional connectivity between highly vascularized areas may be artificially large due to increased signal-to-noise-ratio (SNR) (Vigneau-Roy et al., 2014). Many parameters, such as the correlation threshold and cluster size, can influence functional connectivity matrices (Gorgolewski et al., 2013; Stevens et al., 2013; Woo et al., 2014). On the other hand, structural connectivity based on streamline tractography is known to be biased by the many stopping criteria involved in such reconstructions (i.e., step size, tracking mask, angular deviation, seeding strategy, etc.) (Girard et al., 2014) or algorithms (Bastiani et al., 2012). A slight change in these traditional thresholds might perturb the connectivity profile of certain brain areas. As such, different regions of the brain may benefit from different reconstruction parameters (Chamberland et al., 2014).

Additionally, it can be difficult to select the appropriate rs-fMRI seed points in subjects with pathological developments such as cerebral tumors, lesions, and other abnormalities (Griffa et al., 2013; Chamberland et al., 2014), where white matter bundles and associated cortex are often displaced. In a clinical setting, it would be advantageous if neurosurgeons could instantly view how a slight change in reconstruction parameters impacts the results. Clearly, addressing this issue is difficult, as it would require computing structural and functional connectivity using many sets of pre-defined parameters and then find ways to interact and interpret the connectivity profiles. Therefore, the possibility of visualizing and quantifying structural and functional connectivity while simultaneously modifying important reconstruction parameters could change the way structure-function relationships are studied in single subjects and could lead to an optimized and more efficient way of analysing data in large cohorts of healthy and/or patient populations. Moreover, efficient scientific visualization is important when analysing and illustrating multi-modal MRI data (Irimia et al., 2012; Margulies et al., 2013; Rojas et al., 2014). With emerging human connectome studies and the growing interest of applying rs-fMRI in surgical planning and other clinical applications (Daducci et al., 2012; Griffa et al., 2013; Meskaldji et al., 2013), interactively exploring the circuitry of the brain is essential.

Overall, traditional ways of coupling dMRI with fMRI often come down to reconstructing fiber pathways between distant fMRI regions. However, not much attention has been given in the development of new methods where dMRI assists fMRI. To the best of our knowledge, no previous literature shows how tractography could serve as a guide to generate rs-fMRI connectivity. In this work, we propose an interactive method for the exploration of single-subject brain connectivity in a fully 3D interactive fashion, which can be coupled with our existing real-time fiber tractography method robust to crossings implemented in a freely available software, i.e., the *Fibernavigator*^1^ (Chamberland et al., 2014). Using a new reconstruction technique, namely *tractography-driven resting-state*, we demonstrate how structural and functional connectivity can be merged together to explore the brain in a mutual and interactive manner. The contributions of this work are thus three-fold:

1. We present an interactive method to explore and visualize tractography-driven resting-state functional connectivity.
2. We demonstrate that structural and functional reconstruction parameters may explain a portion of intra- and inter-subject variability.
3. We qualitatively demonstrate how the proposed methods can be used in a neurosurgical planning context.

## 2. Methods

This section is organized as follow: First, we describe the MRI acquisition protocols as well as the data processing. Next, we present the technical implementation and characteristics of the proposed method. Finally, the last part of this section includes the full description of the experiments performed to evaluate the proposed method.

### 2.1. MRI acquisition

Datasets were obtained from 10 young healthy volunteers (ages 21–30, 4 females). In addition, 1 dataset was acquired from a tumor patient (31 year old, male) with astrocytoma of grade III located near the motor cortex. Imaging was performed on a 1.5 T SIEMENS Magnetom (Vision). Subject motion was minimized using head cushions. MRI sessions started with a T1-weighted 1 mm isotropic MPRAGE (TR/TE 1860/3.54 ms) image. Continuous functional recordings were carried out using a standard echo-planar imaging (EPI) sequence (eyes closed). For each run, 108 functional volumes consisting of 35 axial slices were obtained with a 64 × 64 matrix, field of view (FOV) 220 mm, TR/TE 2730/40 ms, for a voxel size of 3.4 × 3.4 × 4.2 mm^3^. Additionally, high angular resolution diffusion imaging (HARDI) data was acquired using a single-shot EPI spin echo sequence (TR/TE = 11700/98 ms), with *b*-value of 1000 s/mm^2^ and 64 uniform directions (matrix size: 128 × 128, 2 mm isotropic spatial resolution). To reduce susceptibility distortions, GRAPPA parallel imaging was employed with an acceleration factor of 2. The study was performed according to the guidelines of the Internal Review Board of the Centre Hospitalier Universitaire de Sherbrooke (Comité d'éthique de la recherche sur I'humain du CHUS).

### 2.2. Data processing

#### 2.2.1. T1 processing

Non-local means (NLM) denoising was applied to the T1-weigthed image prior to using the brain extraction tool (BET) of FSL (Smith, 2002). This facilitated the registration procedure to the upsampled (1 mm isotropic resolution) *b* = 0 diffusion image using ANTS (Avants et al., 2009).

#### 2.2.2. dMRI processing

NLM denoising was performed on the raw diffusion data (Descoteaux et al., 2008). Diffusion tensors, RGB map and corresponding fractional anisotropy (FA) were estimated using MRtrix (Tournier et al., 2012). The single fiber response function was estimated (FA > 0.7). This response function was used as input to spherical deconvolution (Tournier et al., 2007; Descoteaux et al., 2009) to compute the fiber orientation distribution function (fODF) at each voxel of the brain. In this work, we used the efficient implementation publicly available in MRtrix (Tournier et al., 2012) with a maximal spherical harmonics order of 8 and the default parameters. All dMRI derived metrics were upsampled to a 1 mm isotropic resolution using trilinear interpolation (Dyrby et al., 2011; Girard et al., 2012; Smith et al., 2012; Tournier et al., 2012). Finally, the spherical harmonics peaks (i.e., main directions of diffusion, 3 per voxel) of each fODF were then extracted and served as input for real-time fiber tractography (Chamberland et al., 2014).

#### 2.2.3. fMRI processing

Images were first motion and slice-time corrected using the efficient implementation publicly available in AFNI (Cox, 1996). Next, the data were spatially smoothed using NLM denoising (Coupe et al., 2008; Bernier et al., 2014) implemented in Dipy (Garyfallidis et al., 2014) using default parameters, and band-pass filtered (0.008–0.08 Hz). The global signal at each voxel was not regressed to avoid the introduction of anti-correlated regions (Saad et al., 2012).

### 2.3. Resting-state connectivity and visualization

Our interactive rs-fMRI exploration method is implemented on CPU and runs on a single core computer, which does not require any specific hardware. It works on any fMRI data (e.g., resting-state) which is preferably pre-processed (i.e., motion and slice time corrected, spatial and temporal filtered). For anatomical reference, the user has to provide a subject-specific underlying anatomical image (e.g., T1, T2, FA, b0, etc.). By placing a cubical or spherical ROI within the 3D environment (high resolution anatomical space), one can instantaneously activate the functional correlation module while dragging the seed-ROI anywhere in the brain. The mean BOLD signal is first extracted from the voxels encompassed by the ROI, and then statistically compared to the rest of the brain. The correlation coefficient (*r*) between voxels x and y is denoted as: *r* = cov(x, y)/σ_*x*_σ_*y*_, where cov(x, y) is the covariance of the preprocessed BOLD signals and σ_*x*_σ_*y*_ are the standard deviations. The generated correlation coefficients are then converted to *z*-scores (Whittingstall et al., 2013) and rendered at each voxel as small particles (*z* > 0), which are depth-sorted in real-time according to the user's viewing axis.

**Figure 1:**
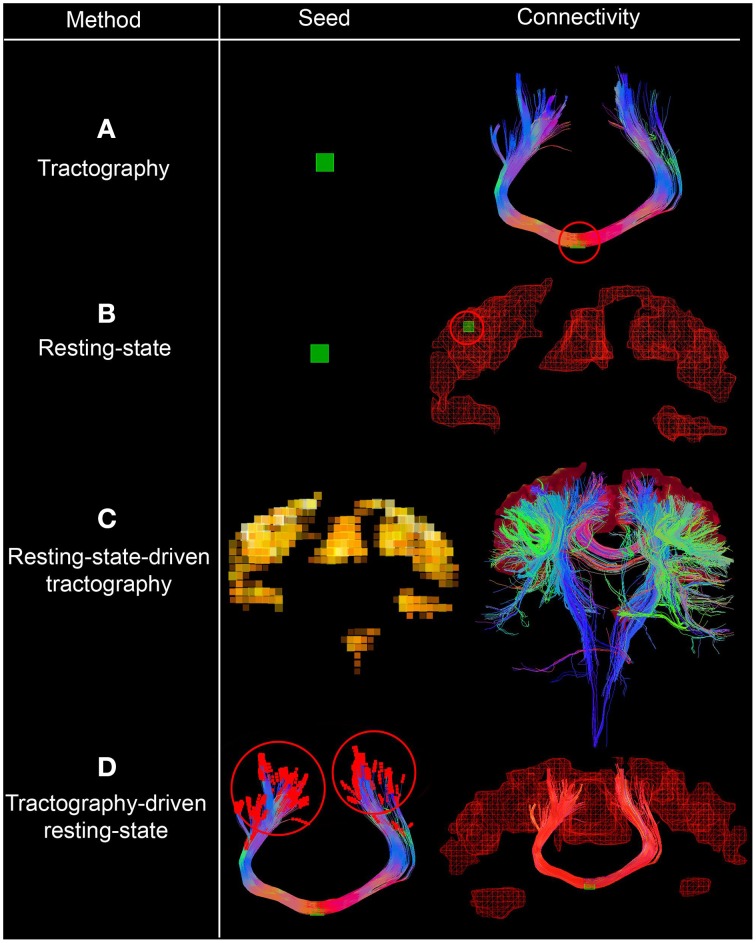
**Interactive seeding methods proposed for structural and functional connectivity**. In **(A,B)**, one can see the traditional approaches for generating structural and functional connectivity using a seed-ROI (green box, circled red on the right). **(C)** Shows the use of functional connectivity for the generation of underlying structural connections by initiating tractography from each rs-fMRI cluster. **(D)** Shows a *streamline-specific* map revealing the underlying functional connectivity associated to the bundle of interest (i.e., corpus callosum, red). Note how functional connectivity extends far beyond the white matter seed points red circles. Supplementary video available online at: www.youtube.com/watch?v=eHSyf2AjbHw.

To reduce cluttering, the opacity (alpha) and size of each particle are weighted by their associated *z*-score value as seen in Figure 1C. This way, regions showing higher correlations are displayed predominantly over less correlated ones. Interactive correlation (*z*-score) and cluster-size (η_*min*_) thresholds are also available for visualization purposes. A flood fill algorithm is responsible for determining the minimum number of connected voxels (faces touching) to form and display fMRI-clusters (with respect to η_*min*_). In a last step, the user can save and export the generated activation map into a 3D nifti file. Note that the computation step is performed in native space (i.e., fMRI space) while the rendering stage is done at the anatomical level (e.g., T1-space) using the scaling transformation matrix associated to the anatomical and rs-fMRI datasets. Therefore, the anatomical and rs-fMRI datasets must be centered at the same origin to ensure proper link between the computation and visualization stages.

### 2.4. Combining functional and structural connectivity

Figure 1 shows the different seeding options proposed to generate and visualize brain connectivity. In Figures 1A–C, one can see the traditional approaches for generating structural and functional connectivity using a seed-ROI (green box). From this ROI, one can generate structural connectivity (i.e., tractography Chamberland et al., 2014, Figure 1A) or functional connectivity (resting-state, Figure 1B). One can also initiate streamlines from functional RSNs to visualize their underlying structural connections (resting-state-driven tractography, Figure 1C). Figure 1D shows a unique method to generate rs-fMRI connectivity by extracting the average BOLD signal from the last *n* points of each streamline, which is then correlated with the rest of the brain (i.e., tractography-driven rs-fMRI). This technique produces a *streamline-specific* map revealing the underlying functional connectivity associated to the bundle of interest. This is further described in the following section.

### 2.5. Experiments

#### 2.5.1. Resting-state networks validation

The first step toward assessing the validity of our interactive correlation method is to compare its results with traditional methods. Using three overlap metrics (defined in the next paragraph), we statistically compared *z*-score maps generated by our real-time technique implemented in the *Fibernavigator* with the ones generated offline using AFNI (Cox, 1996). To do so, we first generated seven commonly reproducible RSNs (Beckmann et al., 2005), namely the default mode network (DMN), motor network, visual network, salience network, lateralized networks and auditory network, by interactively positioning a 10 × 10 × 10 voxels seed-ROI in seven associated anatomical regions (posterior cingulate cortex (PCC), primary somatosensory cortex, primary visual cortex, insula, lateral frontal cortex and primary auditory cortex, respectively). Anatomical localization (MNI space) of the seed regions can be found in Table A1.

The same experiment was performed using AFNI's *3dfim*+ command (Cox, 1996), with the same seed-ROIs as input and converting the resulting correlation coefficients to *z*-scores. The generated RSNs were then statistically thresholded at different conventional *z*-scores (i.e., from 3.0 to 5.0) for statistical comparison. First, the Dice coefficient (D) (Dice, 1945), as used in previous rs-fMRI studies (Kristo et al., 2014; Tie et al., 2014), is a similarity index that allows the quantification of the spatial overlap between two datasets F and G, and is defined as follows: D = 2 ∣F∩G∣ /(∣F∣+∣G∣) = 2*a*/(2*a* + *b* + *c*), where *a* is the number of voxels shared by the two datasets, *b* the remaining voxels of F that differs from G, and *c* the voxels that are present in G but not in F. The advantage of using this metric is that it ranges between 0 and 1. Thus, a perfect fit between two *z*-maps will lead to a Dice coefficient of 1, and 0 if there is no overlap at all. Next, the Jaccard coefficient (J) is computed by taking the intersection over the union between two volumes (J = ∣F∩G∣ /(∣F + G∣) = *a*/(2*a*+*b*+*c*)). This metric expresses the relative volume overlap between the same RSN. The last metric is the correlation coefficient (ρ) between the unthresholded *z*-scores maps, which not only determines if there is spatial correspondence, but also compares the *z*-values between the two maps.

#### 2.5.2. Tractography-driven resting-state fMRI

**Figure 2:**
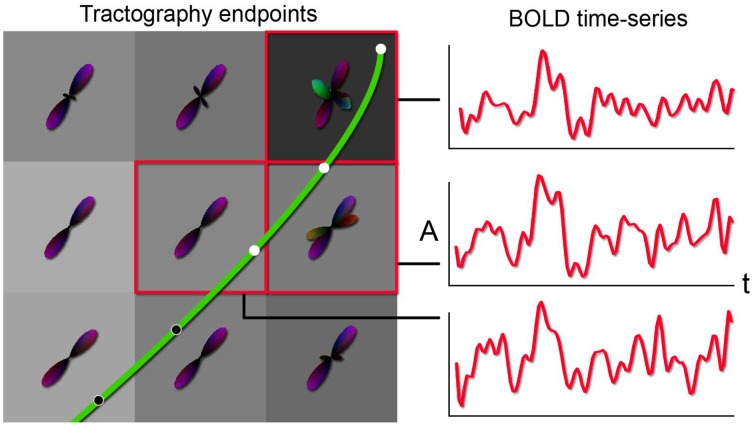
**Principle behind the new tractography-driven rs-fMRI correlation method**. When a streamline reaches the gray matter (left), its propagation is stopped. By looking at the BOLD signals within the voxels encompassed by the last *n* points (3 in this case) of the streamline, the average of the signals is correlated with all other voxels of the brain (right).

Having direct access to interactively adapt the tractography parameters, and more precisely to the stopping criteria (i.e., tracking mask), is a key factor when performing interactive structural connectivity. By interactively positioning a seed-ROI at a specific brain position, the tracking algorithm (Chamberland et al., 2014) performs dense 3D integration along the ODFs-extracted peaks field, thus generating streamlines in a bi-directional fashion. From there on, the 3D coordinates of the last *n* points of each streamline bundle (*n* = 3) were back-projected into fMRI-space to average their underlying BOLD signal and perform real-time correlations with the rest of the brain (Figure 2). Precisely, the mean signal is averaged from all the end points (both sides of the streamlines), unless they terminate outside of the fMRI volume. As the characteristics of the tractography seed-ROI changes (i.e., size, shape and position), the visualization updates. Functional networks associated to the streamlines of interest and their associated tractography parameters are thus revealed. These parameters consist in the tracking mask threshold (τ), the step size (*s*), the angular threshold (θ), the number of seeds (#) and the minimum/maximum streamline length (δ_*min*_∕δ_*max*_). To demonstrate the full potential of the technique, we reconstructed 9 well-known white matter bundles [cingulum (Cg), corpus callosum (CC body, genu and splenium), corticospinal tract (CST), optic radiations (OR), left and right superior longitudinal fasciculus (SLF), and the auditory radiation (AR) Catani and Thiebaut de Schotten, 2012]. Seed-ROIs where interactively positioned at the midbody of each bundle and validated according to co-author and neurosurgeon D. Fortin.

#### 2.5.3. Inter-subject variability

Tractography-driven resting-state was used to demonstrate how parameter selection alone may explain a portion of inter-subject variability typically observed in the DMN (Buckner et al., 2008). Using the *tratography-driven rs-fMRI* method described earlier, we interactively generated the right Cg of a randomly chosen subject (S6) and the underlying associated RSN (i.e., the DMN). Here, reconstruction parameters were set as follows: FA threshold (τ) = 0.15, step size (*s*) = 0.5 mm, maximum angle (θ_*max*_) = 35°, minimum length (δ_*min*_) = 60 mm, maximum length (δ_*max*_) = 200 mm with 1000 seeds evenly distributed within a 4 × 4 × 4 mm ROI located at the mid-coronal section of the Cg body. The rs-fMRI *z*-score and minimum cluster size (η_*min*_) thresholds were 4.0 and 40, respectively. Then, we assessed inter-subject variability by applying those same parameters to the remaining subjects. Next, we regenerated the Cg and associated DMN networks on a individual basis. To achieve this, the reconstruction parameters were interactively set according to the neuroanatomy of each subjects, until the known anatomy of the DMN was retrieved (i.e., functional connectivity in the medial prefrontal cortex (mPFC), the PCC/precuneus, the left and right temporoparietal lobes, Buckner et al., 2008; Ward et al., 2014). The difference and percentage (%) change of each parameter were then computed, defined as follows: (*x* - *y*) for the difference and ((*x* − *y*) / *y* × 100) for the % change, where *x* represents the subject-specific parameters and *y* the reference parameters extracted from S6.

## 3. Results

### 3.1. Resting-state networks validation

**Figure 3:**
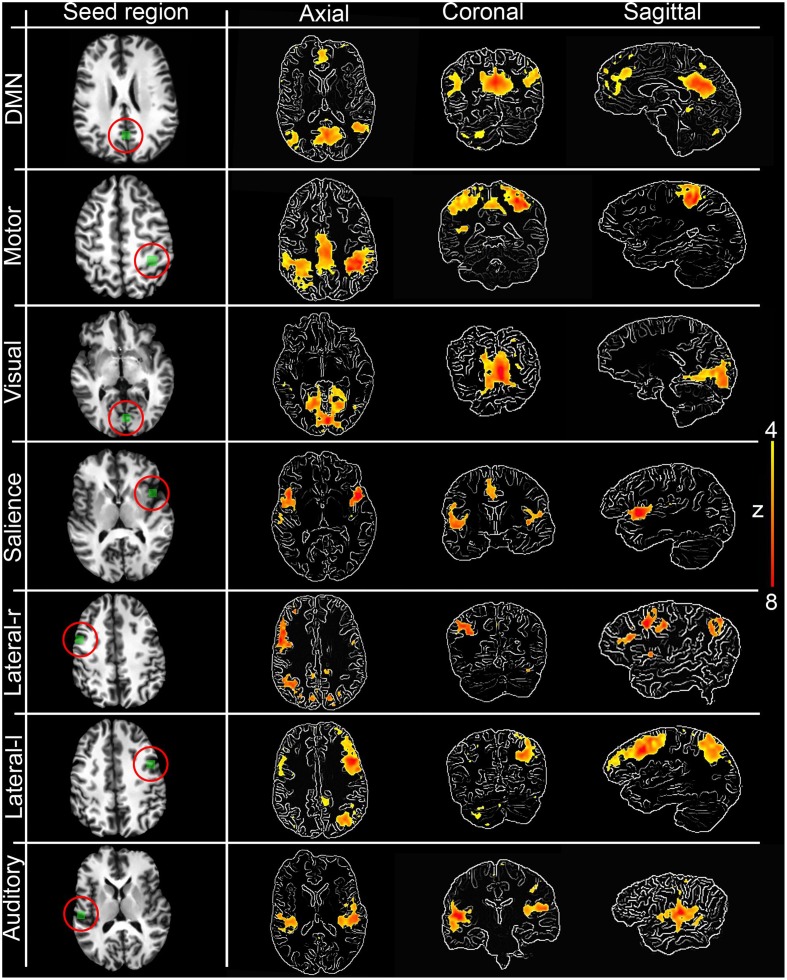
**Interactively generated resting state networks (*z*>4.0) from a single subject used for comparison**. The 3D maps were generated by interactively placing a seed-ROI in the circled (red) anatomical regions. DMN: PCC, Motor network: primary somatosensory cortex, Visual network: primary visual cortex, Salience network: insula, Lateralized networks: lateral frontal cortex and Auditory network: primary auditory cortex. RSNs are overlayed over edge-detected T1 anatomical image for visualization purposes. Anatomical localization (MNI) of the seed regions can be found in Table A1.

Figure 3 shows the seven RSNs used for comparison. As seen in Tables 1, 2, the overlap metrics D, J and ρ, ranged between 0.691 and 0.983 for all RSNs indicating a good overlap regardless of the *z*-score threshold (i.e., *z*>3.0, 4.0, and 5.0). The main finding here is that our real-time interactive implementation is equivalent to offline seed-based rs-fMRI analysis.

**Table 1:** **Comparison of seven RSNs generated with our method with an offline method**.

**RSNs**	**D**			**ρ**
	***z* > 3.0**	***z* > 4.0**	***z* > 5.0**	
DMN	0.957	0.964	0.960	0.983
Motor	0.903	0.925	0.923	0.976
Visual	0.928	0.931	0.925	0.978
Salience	0.914	0.891	0.895	0.977
Lateral-l	0.938	0.935	0.953	0.980
Lateral-r	0.928	0.890	0.825	0.980
Auditory	0.887	0.821	0.817	0.976

Values represent the Dice overlap coefficients (D) for multiple z-scores and a correlation metric (ρ) between the unthresholded maps. Analysis was performed on a single subject (S2) which was randomly selected.

**Table 2:** **Comparison of seven RSNs generated with our method with an offline method**.

**RSNs**	**J**		
	***z* > 3.0**	***z* > 4.0**	***z* > 5.0**
DMN	0.917	0.931	0.923
Motor	0.823	0.861	0.857
Visual	0.866	0.870	0.861
Salience	0.842	0.803	0.811
Lateral-l	0.883	0.879	0.911
Lateral-r	0.865	0.802	0.703
Auditory	0.796	0.697	0.691

Values represent the Jaccard overlap coefficients (J) for multiple z-scores. Analysis was performed on a single subject (S2) which was randomly selected.

### 3.2. Tractography-driven resting-state fMRI

**Figure 4:**
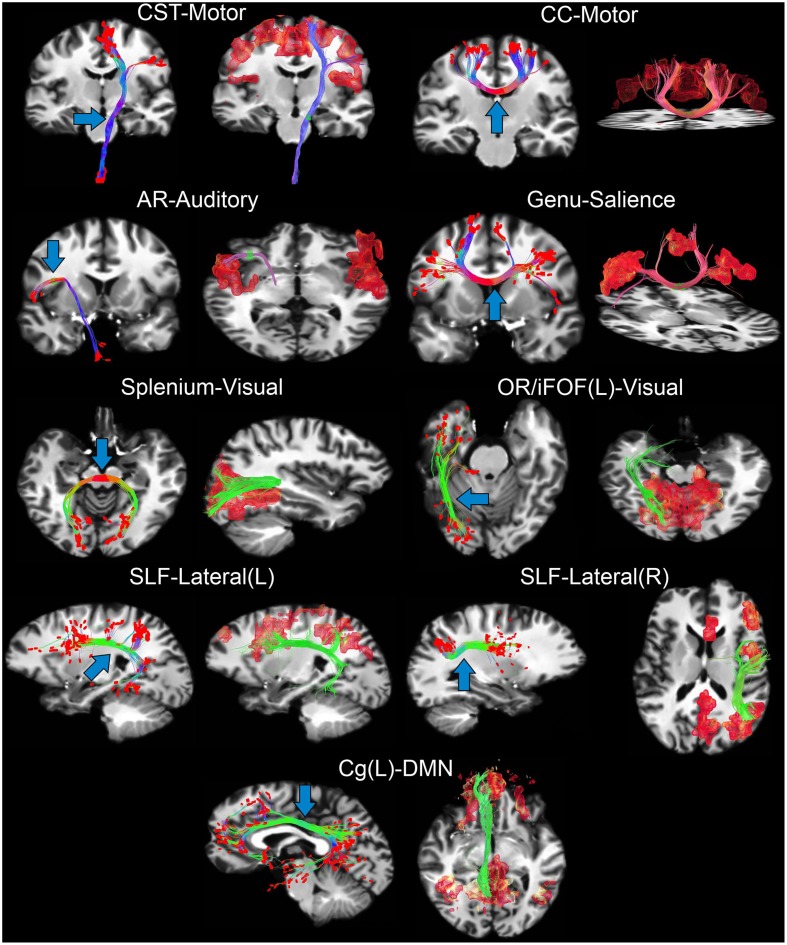
**Tractography-driven resting-state connectivity of 7 RSNs**. Using the last 3 points (red dots) of each streamline (left of each sub-figure), the underlying BOLD signal is extracted, averaged and correlated with the rest of the brain (right of each sub-figure). In order of appearance: CST and the motor network (*z* > 4.8), CC-body and motor network (*z* > 4.9), AR and the auditory network (*z* > 4.0), CC-genu and the salience network (*z* > 4.2), CC-splenium and the visual network (*z* > 5.0), OR-left and the visual network (*z* > 4.4), left and right SLF for the lateralized networks (*z*>4.6), Cg and the DMN (*z* > 4.1). Blue arrows show where tractography seed-ROI was positioned. Note how functional connectivity extends far beyond the white matter seed points for the motor, auditory and DMN networks.

In this section, we present the results of our interactive way of computing rs-fMRI connectivity profiles, based on the extraction of the BOLD signal from the end points of the streamlines reconstructed by tractography. Bundle-specific tractography parameters and associated RSNs are shown in Table 3. Figure 4 shows 9 selected fiber bundles and their end points used to structurally reconstruct rs-fMRI connectivity, namely the Cg and the DMN (*z*-score > 4.1), the CC-midbody and motor network (*z*-score > 4.9), the CST and the motor network (*z*-score > 4.8), the OR-left and the visual network (*z*-score > 4.4), the CC-splenium and the visual network (*z*-score > 5.0), the CC-genu and the salience network (*z*-score > 4.2), the left and right SLF for the lateralized networks (*z*-score > 4.6) and the auditory radiations (AR) underlying the auditory network (*z*-score > 4.0). Minimum cluster level for all RSNs was set to η_*min*_ = 40 voxels (fMRI-space). Tractography seed-ROIs were interactively placed at the midbody of each bundle, indicated by the blue arrows in Figure 4.

**Table 3:** **Bundle-specific tractography parameters used for each RSN**.

**Bundle**	**RSN**	**τ**	***s***	**θ**	**#**	**δ_*min*_**	**δ_*max*_**
Cg (L)	DMN	0.20	1.0	30	1625	100	200
CC (Midbody)	Motor	0.21	1.3	30	638	90	200
CST	Motor	0.20	1.0	25	854	135	200
OR (L)	Visual	0.30	1.0	40	100	100	105
Splenium	Visual	0.15	1.0	35	411	150	200
Genu	Salience	0.20	1.0	28	358	105	135
SLF (L)	Lateral (L)	0.20	1.0	35	284	85	130
SLF (R)	Lateral (R)	0.20	1.0	30	410	60	120
AR	Auditory	0.20	1.4	26	75	105	115

Tractography parameters are as follows: τ, FA threshold; s, step size; θ, maximum angle; #, number of streamlines; δ_min_, minimum streamline length; δ_max_, maximum streamline length. Analysis was performed on subject 2 (S2) which was randomly selected.

Tractography and rs-fMRI parameters where intentionally different for diverse regions of the brain to demonstrate the importance of having region-based parameters to explore structure-function relationship and intra-subject variability (Thiebaut de Schotten et al., 2011). These results show that some functional networks are linked by either direct structural connectivity (Figure 4 motor, salience, visual, lateral networks) or indirect structural connectivity (Figure 4 DMN, lateral networks) (van den Heuvel et al., 2009).

### 3.3. Inter-subject variability

**Figure 5:**
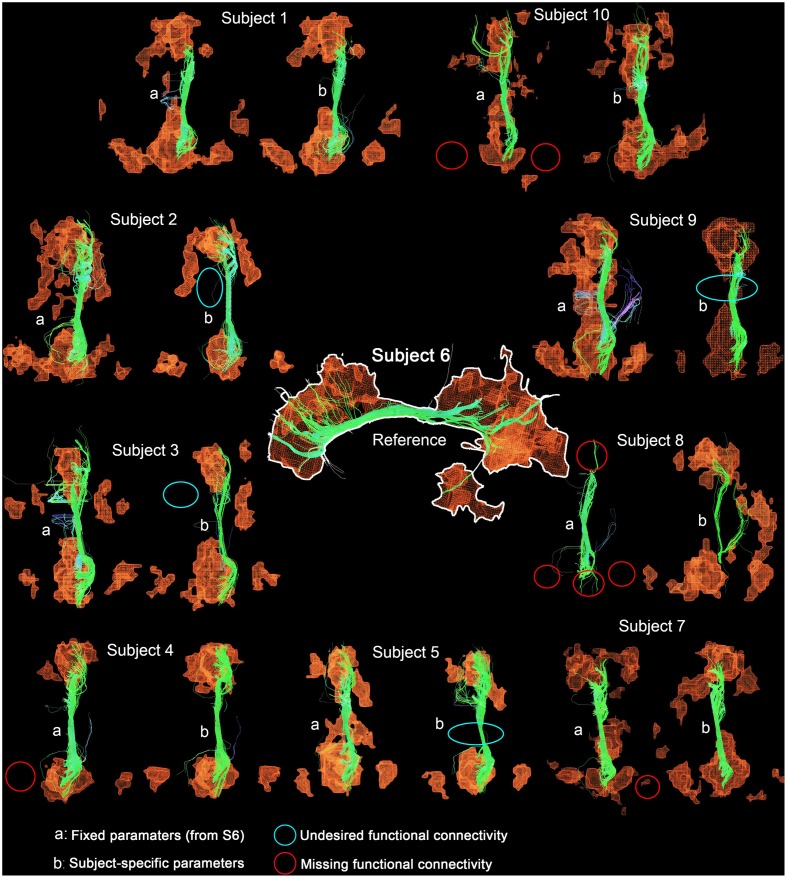
**DMN and Cg (right) displayed for all subjects**. Middle figure shows the network obtained from reference subject (S6) using parameters in Table 4. Applying these parameters to all other subjects yielded networks with annotation **(A)**. Interactively tuning subject-specific parameters (Table 4) produced the **(B)** version of the network. Red circles indicate example regions, where one of the expected nodes could not be retrieved. Blue circles show false-positive clusters successfully suppressed.

Table 4 shows Cg-specific tractography parameters and rs-fMRI *z*-score thresholds used to extract the DMN across 10 subjects. Note the large variability, particularly in the maximum angle (θ) and in the minimum streamline lenth (δ_*min*_). The difference and percentage (%) change of each parameter are displayed in Table 5. The % changes in comparison with reference S6 range from −13.3 to 73.3% for the FA threshold, 20–140% for the step size, ±42.9% for the angle threshold, 0–78.3% for the minimum length and ±20% for the *z*-score threshold. Figure 5 shows the reconstructed DMN and Cg for S6 (middle). Applying these subject-specific parameters on other subjects yielded a Cg bundle and DMN map that varied dramatically (Figure 5A, left representation of each subject). However, when interactively adjusting the tractography and rs-fMRI reconstruction parameters in a subject-specific manner, both the DMN and Cg were easily retrieved (Figure 5B, right representation of each subject). Red circles indicate example regions where one of the expected nodes could not be retrieved. Blue circles shows false-positive clusters successfully suppressed.

**Table 4:** **Cingulum-specific tractography parameters and rs-fMRI *z*-scores used for the assessment of inter-subject variability**.

**Subjects**	**τ**	***s***	**θ**	**δ_*min*_**	**δ_*max*_**	***z*-score**
S1	0.13	0.6	40	90	200	4.20
S2	0.20	1.0	30	100	200	4.10
S3	0.16	1.2	30	107	200	4.20
S4	0.20	1.0	35	90	200	4.00
S5	0.15	0.8	50	110	200	4.60
S6	0.15	0.5	35	60	200	4.00
S7	0.20	1.2	31	80	200	4.05
S8	0.13	0.7	30	160	200	3.20
S9	0.19	1.0	20	60	200	4.80
S10	0.26	0.6	45	100	200	4.00
Avg.	0.18	0.9	35	96	200	4.12
Std.	0.04	0.3	9	29	0	0.42

Parameters: τ, FA threshold; s, step size; θ, maximum angle; δ_min_, minimum streamline length; δ_max_, maximum streamline length (fixed); z-score, minimum rs-fMRI Z threshold. (Avg: average, Std: standard deviation).

**Table 5:** **Difference between reconstruction parameters across subjects, using S6 as reference**.

**Subjects**	**Difference and (% change)**									
**Parameter**	**S1**	**S2**	**S3**	**S4**	**S5**	**S6 (ref.)**	**S7**	**S8**	**S9**	**S10**
τ (unit free)	−0.02 (−13.3)	0.05 (33.3)	0.01 (6.7)	0.05 (33.3)	0 (0)	−	0.05 (33.3)	−0.02 (−13.3)	0.04 (26.7)	0.11 (73.3)
*s* (mm)	0.1 (20)	0.5 (100)	0.7 (140)	0.5 (100)	0.3 (60)	−	0.7 (140)	0.2 (40)	0.5 (100)	0.1 (20)
θ (°)	5 (14.3)	−5 (−14.3)	−5 (−14.3)	0 (0)	15 (42.9)	−	−4 (−11.4)	−5 (−14.3)	−15 (−42.9)	10 (28.6)
δ_*min*_ (mm)	30 (50.0)	40 (66.7)	47 (78.3)	30 (50.0)	50 (83.3)	−	20 (33.3)	100 (166.7)	0 (0.0)	40 (66.7)
*z*-score (unit free)	0.2 (5.0)	0.1 (2.5)	0.2 (5.0)	0 (0.0)	0.6 (15.0)	−	0.05 (1.3)	−0.8 (−20.0)	0.8 (20.0)	0 (0.0)

The results from Tables 4, 5 and Figure 5 show that the parameters needed to reconstruct the Cg varies dramatically across subjects. As a result, so does the DMN. Differences present in connectivity profiles show the importance of having subject-specific parameters when looking at structure-function relationships. Other parameters such as the number of seeds and the minimum rs-fMRI cluster size remained fixed.

### 3.4. Neurosurgical planning application

**Figure 6:**
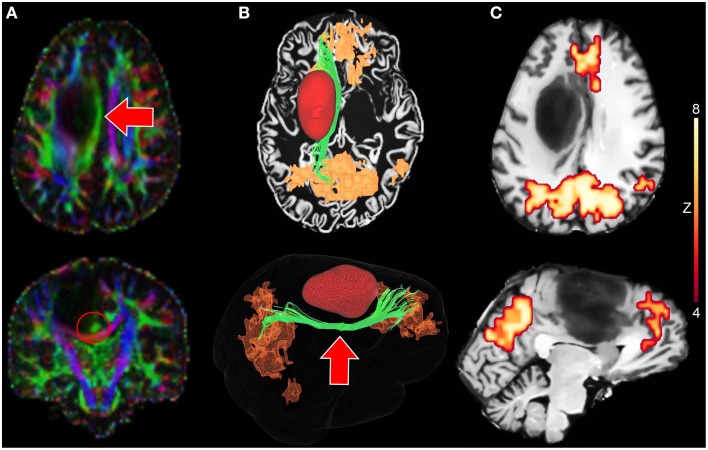
**Tractography-driven reconstruction of the DMN applied to a neurosurgical case**. **(A)** Axial and coronal views of RGB map showing the deviated Cg (red arrows) induced by the mass effect of the tumor. **(B)** 3D reconstruction of the Cg (green), the tumor (red) and the DMN (orange) based on tractography-driven resting-state. Seed-region was positioned at the mid-body of the Cg (red circle). **(C)** 2D axial and sagittal views of DMN-overlayed T1 map showing functional connectivity near the tumor (mPFC).

The proposed interactive functional and structural reconstruction methods were introduced at the pre- and intra-operative levels for a neurosurgical intervention. The case consists of a 31 years old male tumor patient with astrocytoma of grade III located near the motor area. Figure 6 shows a tractography-driven reconstruction of the DMN. Axial and coronal views of RGB map (Figure 6A) show the deviated Cg (red circle) induced by the mass effect of the tumor. 3D reconstructions of the Cg (green), tumor (red), and the DMN (orange) are illustrated in Figure 6B. Figure 6C reveals functional connectivity near the anterior side of the tumor (mPFC). The Cg was reconstructed using the following standard tractography parameters: min. FA = 0.2, *s* = 0.5 mm, max. θ = 25°, δ_*min*_ = 90 mm, δ_*max*_ = 130 mm. The rs-fMRI *z*-score and cluster-size thresholds were set to 4.2 and 20, respectively. These parameters are comparable to state-of-the-art reconstruction parameters used for healthy subjects (Tables 3, 4) (Castellano et al., 2012; Tournier et al., 2012). The DMN was then generated by performing the temporal correlation of the BOLD signal underlying the last 3 points of each streamlines with all the other voxels of the brain.

**Figure 7:**
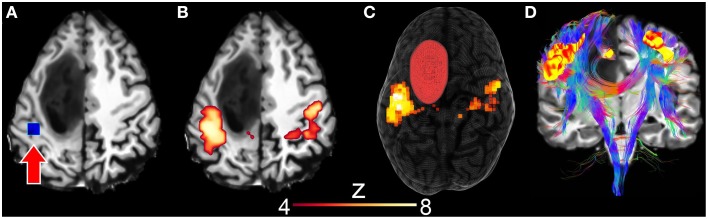
**Surgical application using resting-state driven tractography to uncover the functional and structural motor network of a 31 years old tumor patient with astocytoma of grade III**. **(A)** Seed-ROI (blue box, red arrow) interactively positioned in the motor cortex. **(B)** 2D correlation map revealing the motor network (*z* > 4.0). **(C)** 3D rendering of segmented tumor and motor RSN. **(D)** Underlying white matter fiber pathways (CST, CC) generated using 28,000 seeds evenly distributed within the previously described rs-fMRI motor cluster map.

Next, using *resting-state driven tractography*, we performed the instantaneous reconstruction of white matter fiber pathways using rs-fMRI clusters as seen in Figure 7. First, the motor RSN was generated by interactively positioning a 10 × 10 × 10 mm seed-ROI within the motor cortex (Figures 7A–C, *z*-score > 4.0). Next, tractography was initiated by evenly distributing 28000 seeds (1 seed per voxel) within the uncovered motor network clusters, thus allowing the reconstruction of the motor pathways (Figure 7D). Tractography parameters were set as the following: min. FA = 0.2, *s* = 1.0 mm, max. θ = 35°, δ_*min*_ = 10 mm, δ_*max*_ = 200 mm. Generating such a connectivity profile offline would require of the user to (1) “blindly” select a seed-ROI and (2) observe the associated connectivity map before (3) using it as a seeding mask for tractography. One could also use independent component analysis (ICA) (Beckmann et al., 2005; Damoiseaux et al., 2006), which is another way of extracting multiple RSNs of the brain by statistically decomposing the acquired signal in a set of separate intrinsic components. It then still requires the appropriate selection of the component associated to the motor network, to finally inject it as a seeding mask for tractography.

**Figure 8:**
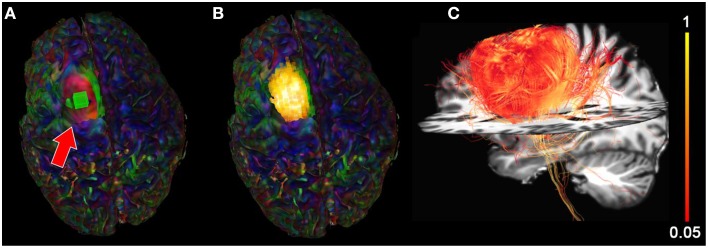
**Functional segmentation of a brain tumor using the BOLD signal**. **(A)** rs-fMRI seed-ROI (greenbox, red arrow) interactively placing within the tumor area. **(B)** 3D voxels (*z*-score > 5.0) showing strong functional correlation within the tumor. **(C)** Fiber tractography generated using the functionally-segmented tumor mask showing structural organization in the vicinity of the tumor. Colormaps: **(A,B)** RGB, **(C)** FA.

We then extracted the BOLD signal of the tumor area by placing a 10 × 10 × 10 mm seed-ROI inside the tumor region (Figure 8A) resulting in a functional segmentation of the astrocytoma (Figure 8B). Next, using the functionally-driven map (dilated by 3 mm to cover the boundaries of the WM), tractography was initiated by interactively lowering the tracking threshold (FA) from conventional values (0.10−0.20, Castellano et al., 2012) to 0.08. This enabled the reconstruction of “low-FA” streamlines surrounding the tumor, as shown on Figure 8C. These streamlines, comprised the CST and CC, wrapped laterally around the tumor. Supplementary material available online at www.youtube.com/watch?v=eHSyf2AjbHw illustrates the real-time interactive methods introduced in this section.

## 4. Discussion

In this study, we proposed a new interactive method to generate tractography-driven resting-state functional connectivity which generates bundle-specific functional networks. We also show the problems associated with the use of fixed reconstruction parameters across different brain regions and individuals. Our experiments reinforce the idea of using region- and subject-specific parameters based on the neuroanatomy of each individual. Finally, the proposed methods were applied in a neurosurgical context, which allowed the exploration of structure-function relationships to achieve *subject-specific medicine*.

### 4.1. Visualization and interactivity

Interactively coupling structural and functional imaging can provide great insights about brain connectivity, especially with the need to use rs-fMRI in surgical planning and other clinical applications, and the advent of human connectome studies. Some have proposed a tool for voxel-wise brain connectivity visualization, but the method requires the pre-calculation of a voxel-by-voxel correlation matrix (Dixhoorn et al., 2012) which can be hundreds of Gigabytes. In addition, GPU implementations have been developed for functional connectivity exploration (Eklund et al., 2011, 2013) or for pre-surgical planning (Böttger et al., 2011). The proposed methods, however, restricts the user from placing a seed-ROI at any point in the 3D space, which greatly reduces the level of interactivity. Subsequently, the user is limited to move the seed-ROI solely on 2D anatomical slices, thus only revealing activations present on displayed slices. Moreover, none of the aforementioned methods proposes the fusion of both rs-fMRI and dMRI data. The efficient AFNI toolbox (FATCAT) does propose a method for combining rs-fMRI and dMRI, but demands the precomputing of whole-brain tractography with fixed parameters (Cox, 1996; Saad and Reynolds, 2012; Taylor and Saad, 2013). In addition, the proposed exploration tool does not allow direct superposition of RSNs and underlying streamlines, as the 3D brain hemispheres must be separated to achieve proper visualization.

In this paper, the mean frame-per-second (FPS) index is over 20 when solely activating the real-time tractography method with default parameters (anatomical space, 1000 seeds) (Chamberland et al., 2014). The same interactivity level (30+ FPS) applies for rs-fMRI connectivity, even when setting the *z*-score threshold to 0 (thus displaying thousands of correlation factors). For higher resolution resting-state acquisition (e.g., 3T scanner, assuming a 3 mm^3^ voxel size, 250 volumes, TR ≃ 2 s, 8 min total), the interactivity would decrease in a “soft” real-time fashion. This means that as the number of time-points increase, the FPS index decreases. One potential solution would be to implement the correlation step on GPU (Eklund et al., 2011, 2013). This would also allow the use of advanced techniques such as time-lag and partial correlation. For tractography-driven rs-fMRI, the interaction remains fluid as long as the maximum number of tractography seeds within the interactive ROI does not exceed 1000. When performing resting-state driven tractography, these 1000 seeds must thus be shared between the displayed fMRI clusters to maintain a high level of interactivity while moving the seed-ROI.

To the best of our knowledge, our proposed approach is the first study to interactively perform rs-fMRI connectivity based on tractography results. We strongly encourage the reader to visualize the following video to fully appreciate the contributions of this work and the necessity of an interactive method: www.youtube.com/watch?v=eHSyf2AjbHw.

### 4.2. Seed-based vs. independant component analysis

Independent component analysis (ICA) is another way of extracting multiple RSNs of the brain by statistically decomposing the acquired signal in a set of separate intrinsic components (Beckmann et al., 2005; Damoiseaux et al., 2006). By doing so, ICA does not require any a priori assumption regarding the selection of a seed-ROI. However, the method is not well-suited for interactive exploration of RSNs due to its intensive mathematical decomposition algorithm, and still requires the selection of the desired networks manually or semi-automatically. Most functional connectivity tools are developed for group analysis, such as group-ICA (Beckmann et al., 2009), where group-components are regressed back to individuals, which can be heavy to use for single subject analysis. In addition, it has also been shown that seed-based and ICA methods provide comparable results when looking at rs-fMRI connectivity (Damoiseaux et al., 2006; Long et al., 2008; van den Heuvel et al., 2008; van den Heuvel and Hulshoff Pol, 2010; Rosazza et al., 2012).

### 4.3. Offline validation with AFNI

The quantitative overlap measures (D and ρ) showed that our real-time interactive method can reproduce reliable networks and is comparable to state-of-the-art offline techniques. More specifically, the DMN, motor, visual, salience, and lateral-l networks showed the highest D overlap and consistency across different *z*-score thresholds (mean ± std, 0.960 ± 0.004, 0.917 ± 0.012, 0.928 ± 0.003, 0.900 ± 0.012, respectively). The lateral-r and auditory networks metrics showed slightly lower D metrics (0.881 ± 0.052, and 0.842 ± 0.034) while maintaining a high ρ factor (0.980 and 0.976). The underlying correlation method is a possible source of variability. Our implementation performs the temporal correlation with all voxels of the brain, and only the positive-valued correlation factors are converted to *z*-score, while the remaining are set to 0.

### 4.4. Tractography-driven resting-state fMRI

Tractography-informed rs-fMRI connectivity is a promising new method that could reveal limits and indirect connections of multiple RSNs (e.g., functional regions that does not share a structural link). Having access to the tractography stopping criterion in real-time allows the instantaneous visualization of the effect of those parameters on the reconstructed networks. Tractography parameters where intentionally different across bundles. This demonstrates the importance of having region-based parameters, thus enabling the exploration of intra-subject variability. The main purpose of using a higher minimum streamline length criteria (δ_*min*_ > 60 mm) than default settings (e.g., 10 mm, Tournier et al., 2012) is to eliminate undesired short streamlines that would otherwise terminate prematurely within the white matter. Further research has to be conducted to see how the location of tractography end points impacts on rs-fMRI connectivity. The proposed technique could benefit from more thorough stopping criteria such as introducing anatomical priors (Smith et al., 2012; Girard et al., 2014), to ensure that all streamlines terminate within the gray matter. In addition, the step size and the number of terminal points per streamline used for correlation are also directly linked. One has to keep in mind that as the step size changes, the number of end points used to perform functional correlation should potentially be adapted accordingly. Interestingly, we also noticed that sometimes even a few streamlines reaching the appropriate cortex region are sufficient to generate a whole RSN. Overall, this method has the advantage of making good use of the tractography parameters by precisely adjusting them according to the desired network of interest. Specifically, this experiment showed that multiple RSNs can be recovered (Figure 4) using this original seeding strategy.

### 4.5. Inter-subject variability

The morphology of the brain substantially differs between individuals (Mueller et al., 2013). Traditional ways of coupling fMRI and dMRI often come down to reconstructing fiber pathways between distant fMRI activation regions. This may be problematic for two reasons: First, it assumes that fMRI should lead to dMRI fiber reconstruction, but it is not always the case. Actually, what do functional connectivity profiles look like when fMRI seed regions are determined via the end points of key white-matter bundles? Secondly, the vast majority of structure-function studies are analysed by assuming fixed reconstruction parameters across all subjects, despite the fact that this has been shown to lead to false-negatives (Chamberland et al., 2014).

We assessed the inter-subject variability associated to the structure-function relationship by importing a set of fixed parameters from an individual to the rest of our subjects. The right Cg bundle of each individual was reconstructed and the last 3 points of each streamline served as seed-ROI to perform correlations with the underlying BOLD signal present at these voxels. This allowed the recovery of the DMN in all cases, but with qualitative differences in their structural and functional connectivity profiles. The fixed set of parameters produced either artificially large functional connectivity spread across the brain, or could not fully recover the expected nodes of the DMN (mostly those located at the temporoparietal junction, Figure 5). Table 5 revealed differences in subject-specific parameters ranging from −0.02 to 0.11 for the FA threshold, 0.1–0.7 mm for the step size, ± 15° for the angle threshold, 0–100 mm for the minimum length, and ± 0.8 for the *z*-score threshold. This variability shows the importance of having subject-specific parameters when looking at brain connectivity across multiple subjects. These parameters are manually set based on anatomical knowledge. One could think of a machine-learning method that could qualitatively set each parameter based on the location and characteristics of the seed-regions.

Other adjustments such as the number of seeds, the minimum cluster size η_*min*_ or the ROI's size, shape, and position should not be neglected. One should note that the proposed technique can robustly recover functional connectivity (e.g., DMN) from its underlying structural connectivity (e.g., Cg) across different subjects but can also extend to other well-known RSNs (e.g., motor, auditory, visual, salience, lateralized, and DMN networks, Figure 4).

### 4.6. Application in neurosurgical context

From a neurosurgical perspective, it could be interesting to look at the underlying functional network associated to an intra-operatively stimulated cortical site. This would require the preprocessing of multiple correlation maps associated to every seed-ROI, which may vary in size, shape and position. For subjects with brain tumors, the co-registration with templates is also an issue. Indeed, the use of predefined seed regions can thus be inefficient to explore rs-fMRI data due to distortions induced by the mass effect of the tumor. To overcome this problem, we have introduced an interactive method at both pre- and intra-operative levels. In this way, it is straightforward for the user to place a seed region (with adjustable size, shape and position) at any point of the brain, according to the will of the neurosurgeon. The use of the tractography-driven resting-state method enabled recovery of a functional network underlying a tumor-induced deviated structure (i.e., Cg).

We also showed that the BOLD signal can be used to characterize, and more specifically functionally segment brain tumors, as previously mentioned in the literature (Ulmer et al., 2004; Hou et al., 2006; Feldman et al., 2009). Moreover, the use of this functionally-driven map allowed us to initiate tractography and identify infiltrated streamlines within the surroundings of the tumor. By interactively lowering the tracking threshold in real-time (i.e., FA > 0.08), coherent structure was found. Such structure would not have been reconstructed if a higher FA threshold had been used (e.g., FA > 0.1, Castellano et al., 2012). Therefore, it is of tremendous importance to tune parameters in a subject-specific way also in neurosurgical applications since this leads to more reliable pathway estimates. Hence, neurosurgeries should be individually adapted for the neuroanatomy of each patient. Another potential application of the proposed methods is for pre- and intra-operative planning for patients with epilepsy where mapping the epileptic foci and its surrounding connections is critical (Liu et al., 2009; Otte et al., 2012; Taimouri et al., 2014; Tax et al., 2014). For neuro-degenerative diseases, we believe that our approach is important to more accurately visualize the alternation of a particular RSN. For instance, our software package could be used to freely explore which parameters (correlation threshold, seed location) need to be adjusted to highlight the often-observed DMN. By doing so, the user could then use this information as starting point for group analysis.

## 5. Conclusion

In this paper, we proposed a method for probing functional and structural connectivity in a 3D interactive fashion. By extracting the BOLD time series from the end points of a streamline and performing real-time correlations with the rest of the brain, we demonstrated that multiple well-known RSNs can be recovered. This provides more insight on the structure-function relationship in a subject-specific aspect. It can also serve as a quality assurance technique at the single subject level prior to launching massive analysis. Importantly, researchers should be careful when using fixed parameters across multiple subjects, which potentially rules out most of the inter-individual variability. In conclusion, our proposed method can be used for clinical applications and is achievable without complex GPU programming. Future development will include a more thorough assessment of the white matter and gray matter characterization by adding anatomical priors to the tractography algorithm (Girard et al., 2014). Supplementary video data showing the real-time interactive reconstruction of brain networks can be found online at: www.youtube.com/watch?v=eHSyf2AjbHw.

### Conflict of interest statement

The authors declare that the research was conducted in the absence of any commercial or financial relationships that could be construed as a potential conflict of interest.

## Footnotes

^1^Open source software available online at: www.github.com/chamberm/fibernavigator.

## Appendix

**Table A1:** **Spatial localization in MNI space coordinates of seed regions used for Figure 3**.

**Anatomical label**	**Coordinates (MNI)**
Posterior cingulate cortex	0, 58, 47
Primary somatosensory cortex	40, 24, 69
Primary visual cortex	6, 83, 22
Insula	46, −11, 4
Lateral frontal cortex (left)	45, −20, 42
Lateral frontal cortex (right)	−51, −18, 47
Primary auditory cortex	−55, 22, 22
